# Prediction of Strength and CBR Characteristics of Chemically Stabilized Coal Gangue: ANN and Random Forest Tree Approach

**DOI:** 10.3390/ma15124330

**Published:** 2022-06-18

**Authors:** Muhammad Nasir Amin, Mudassir Iqbal, Mohammed Ashfaq, Babatunde Abiodun Salami, Kaffayatullah Khan, Muhammad Iftikhar Faraz, Anas Abdulalim Alabdullah, Fazal E. Jalal

**Affiliations:** 1 Department of Civil and Environmental Engineering, College of Engineering, King Faisal University, Al-Ahsa 31982, Saudi Arabia; kkhan@kfu.edu.sa (K.K.); 218038024@student.kfu.edu.sa (A.A.A.); 2 Shanghai Key Laboratory for Digital Maintenance of Buildings and Infrastructure, State Key Laboratory of Ocean Engineering, School of Naval Architecture, Ocean & Civil Engineering, Shanghai Jiao Tong University, Shanghai 200240, China; mudassiriqbal29@sjtu.edu.cn (M.I.); jalal2412@sjtu.edu.cn (F.E.J.); 3 Department of Civil Engineering, University of Engineering and Technology, Peshawar 25120, Pakistan; 4 Department of Civil Engineering, National Institute of Technology Warangal, Warangal 506004, India; gmohdashfaq@gmail.com; 5 Interdisciplinary Research Center for Construction and Building Materials, Research Institute, King Fahd University of Petroleum and Minerals, Dhahran 31261, Saudi Arabia; salami@kfupm.edu.sa; 6 Department of Mechanical Engineering, College of Engineering, King Faisal University, Al-Ahsa 31982, Saudi Arabia; mfaraz@kfu.edu.sa

**Keywords:** lime dosage, gypsum, coal gangue, chemical stabilization, ANN model, random forest model

## Abstract

Coal mining waste in the form of coal gangue (CG) was established recently as a potential fill material in earthworks. To ascertain this potential, this study forecasts the strength and California Bearing Ratio (CBR) characteristics of chemically stabilized CG by deploying two widely used artificial intelligence approaches, i.e., artificial neural network (ANN) and random forest (RF) regression. In this research work, varied dosage levels of lime (2, 4, and 6%) and gypsum (0.5, 1, and 1.5%) were employed for determining the unconfined compression strength (UCS) and CBR of stabilized CG mixes. An experimental study comprising 384 datasets was conducted and the resulting database was used to develop the ANN and RF regression models. Lime content, gypsum dosage, and 28 d curing period were considered as three input attributes in obtaining three outputs (i.e., UCS, unsoaked CBR, and soaked CBR). While modelling with the ANN technique, different algorithms, hidden layers, and the number of neurons were studied while selecting the optimum model. In the case of RF regression modelling, optimal grid comprising maximal depth of tree, number of trees, confidence, random splits, enabled parallel execution, and guess subset ratio were investigated, alongside the variable number of folds, to obtain the best model. The optimum models obtained using the ANN approach manifested relatively better performance in terms of correlation coefficient values, equaling 0.993, 0.995, and 0.997 for UCS, unsoaked CBR and soaked CBR, respectively. Additionally, the MAE values were observed as 45.98 kPa, 1.41%, and 1.18% for UCS, unsoaked CBR, and soaked CBR, respectively. The models were also validated using 2-stage validation processes. In the first stage of validation of the model (using unseen 30% of the data), it was revealed that reliable performance of the models was attained, whereas in the second stage (parametric analysis), results were achieved which are corroborated with those in existing literature.

## 1. Introduction

Sustainable development is an aspiration of most developing countries which in principle, deals with the inclusiveness of environmental, societal, and economic concerns of a nation [1,2]. In order to meet this aspiration, there is a need for a paradigm shift in the economic models and policymaking of developing countries. However, the dependence on the traditional method of thermal power production hampers the achievement of sustainability goals [3]. In India, the primary energy source is thermal power which contributes close to 70% of the total energy generated [4,5]. The coal mining operations are widely promoted to address the ever-increasing demand for thermal power [6]. However, during the mineral processing and extraction stages of coal mining operations, vast quantities of waste are generated [7,8]. The procurement and disposal of coal mining waste is a universal problem. Coal gangue (CG) is the largest (20–40%) among the various wastes associated with coal mining operations generated during the processing/washing of coal blocks [9]. Globally, CG is known through various names such as “coalmine stones”, “coal wash”, “coal spoils/tailings” and it is characterized by great variation in grain size and petrographic composition. The large quantities of CG dumps can contribute to existing environmental and ecological issues, and have implications on the health and safety of residents. The CG dump, which is susceptible to weathering and decomposition, may lead to a dust storm and acidification of ground and subsurface water. Further, in tropical climates, it may swiftly lead to collapse, spontaneous combustion, and spray explosion. Among the industrial solid wastes, CG is one of the largest and most harmful wastes generated from the coal production process. Among the existing wastes related to coal mining, extensive studies have been performed on CCRs. However, relatively lesser attention has been focused on CG than that of CCRs. The variation in CG utilization is driven by its physical and chemical heterogeneity [10,11]. In China, the largest consumer of CG, it is extensively utilized in brick production, thermal energy generation, and fill material for land reclamation [12]. In Europe, its application is limited as fill material in earthworks and subbase/subgrade material in pavements [13]. The current usage of CG in India is confined to fill material to reclaim open cast mine sites [14,15,16]. Considering the potential negative impacts of CG dumps, it is essential to identify alternative utilization modes. Recently, there has been a growing impetus to improve the engineering characteristic of CG to facilitate larger utilization. Pre-loading and dynamic compaction methods were proposed by Koutsoftas and Kiefer [17] for enhancing the strength and compression characteristics of CG fills. Davies, M.C. [18], and Okagbue and Ochulor [19] evaluated the influence of cement addition on the engineering characteristics of CG. The effect of compaction in the enhancement of geotechnical properties of CG was identified by Indraratna et al. [10]. The authors established that the compaction of CG facilitates the dissipation of pore pressure which is often manifested in silty sands [7]. A thorough comprehension of environmental implications associated with the current and future approach of CG usage has vital importance. However, industrial byproducts such as CG in their native form may not meet the design specifications of a subbase material in pavements. Traditionally, industrial byproducts are stabilized by lime, cement, and fly ash (self-cementing class C). Earlier studies by Okagbue and Ochulor [11] and Cao et al. [12] noted the cement-stabilized CG exhibited the desired characteristics of a subbase material that can be potentially utilized in low volume roads. The findings were corroborated by Rujikiatkamjorn et al. and Tasalloti et al. [13,14], and Ashfaq et al. [15]; who have inferred that CG engineering properties are favorable to facilitate its application in earthworks. However, the studies on the variation in strength and CBR characteristics of chemically stabilized CG are scarce. Against this background, an effort was made to evaluate the effect of lime and gypsum addition on the unconfined compression strength (UCS) and California bearing ratio (CBR) of CG.

To overcome the limitation of the laborious and time-consuming nature of laboratory studies for the evaluation of engineering characteristics, there is a growing trend to develop numerical models for solving engineering problems [16,17] and prediction models for swift estimation of engineering characteristics of soils. Most prediction models are developed on the basis of regression analysis with a relatively limited database. To overcome this limitation, the advent of artificial intelligence-based models is being promoted primarily due to their ability to estimate/predict results even for larger databases. Sinha and Wang [18] are among the early researchers to introduce artificial neural networks (ANN) a machine learning language of MATLAB for the estimation of compaction characteristics for a database of 55 soil samples. The advent of ANN resulted in its consistent usage in dealing with complex physical and mathematical problems [19,20,21,22,23]. However, currently, there is minimal literature on AI models regarding their application in predicting engineering characteristics of industrial byproducts. Furthermore, the random forest (RF) computation method presents an alternative approach with an enhanced prediction capability and minimal non-overlapping of the developed models for greater accuracy in calculations. This study details the development of ANN and RF models to forecast the strength and CBR characteristics of chemically stabilized CG. Based on the developed model, a parametric and sensitivity analysis was performed to examine the effect of the stabilizer dosage and curing period on the engineering characteristics of CG.

## 2. Materials and Methodology

### 2.1. Laboratory Studies

**Figure 1:**
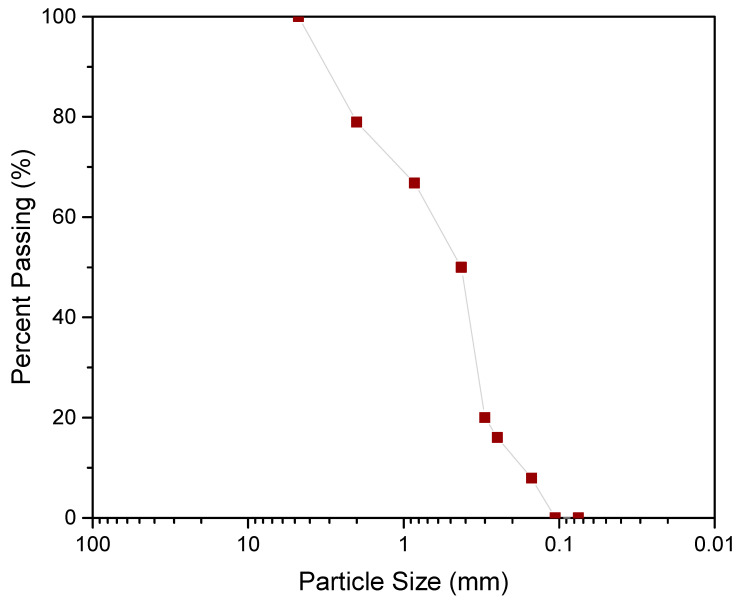
Particle size distribution curve of the crushed coal gangue.

**Figure 2:**
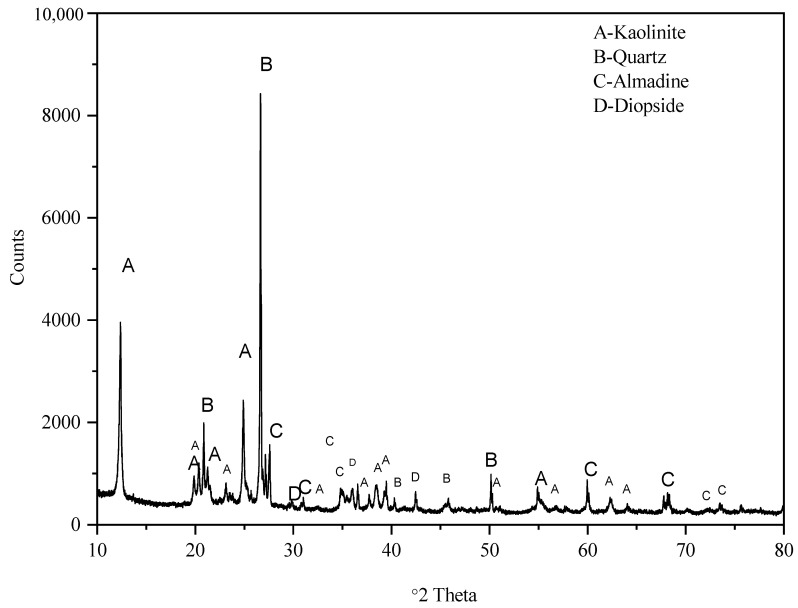
X-ray diffractograms of the coal gangue considered in this study for identification of various peaks of clay minerals.

The CG considered in the current study was acquired from Singareni Collieries (18°25′53.2″ N; 79°51′30.8″ E), Bhupalpally, Telangana, India. For UCS and CBR testing, 425 µm passing fraction was considered according to ASTM provisions and to avert the boundary effects associated with variation in particle size. In Table 1, the CG properties (controlled case) and the adopted ASTM standards are presented. Firstly, the CG samples were subjected to grinding using a jaw crusher, followed by manual crushing, and sieving and efforts to maintain homogenous fines for all the CG samples. The grain size distribution curve presented in Figure 1 corresponds to the 4.75 mm passing fraction whereas Figure 2 depicts the X-ray diffractogram. Prior to testing, the CG samples were statically compacted to 95% of maximum dry density at a strain rate of 0.35 mm/min. To avert the adverse effects of moisture fluctuations, a constant moisture content of 17% was maintained for all the samples. The lime and gypsum dosage of 2, 4, and 6% and 0.5, 1, and 1.5% were varied for different combinations of testing. The lime dosage was fixed based on the initial consumption of lime (ICL) value of 4.2, and the gypsum dosage was limited to 1.5% to avoid the formation of ettringite, a natural swelling mineral [24]. The samples were cured for 7, 14, 21, and 28 days in a temperature-controlled desiccator. The specimens which exhibited excessive hydration (>5% reduction in weight) were not considered for testing. The chemical composition of CG is presented in Table 2. This confirms the observation made in literature on the excessive presence of silica and alumina (>75%) an essential component for pozzolanic reaction.

Initially, six specimens of each were prepared for the control samples cured at 1,7,14, and 28 days, respectively. The percentage of lime dosage varied between 0 and 6%. Hence, the number of samples prepared at this point was 96. Subsequently, the mix was continued similarly except the gypsum dosage was changed from 0 to 0.5, 1, and 1.5% (Table 3), thus yielding total of 384 samples.

### 2.2. Overview of the Developed Model

This section discusses the AI model used in this study. This section also illustrates the hyperparameter used in this study for the development of models.

#### 2.2.1. Artificial Neural Network Model

**Figure 3:**
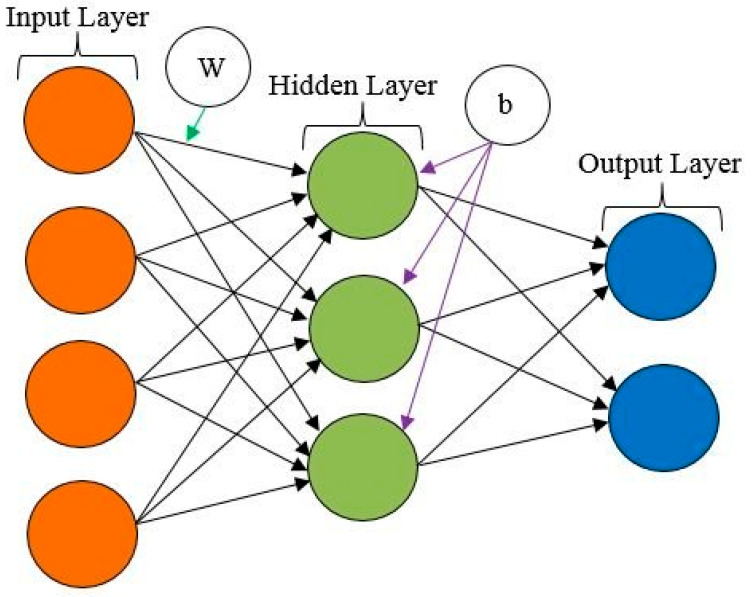
A typical neuron from an artificial neural network (adapted from Rahman, M.A., Muniyandi, R.C., Islam, K.T. and Rahman, M.M. [27]).

Artificial neural networks (ANNs), proven to be effective for data mining applications, are designed after the brain’s biological neurons, which are formed of numerous layers of linked artificial neurons that perform a series of data transformations to arrive at the desired output [25,26,27]. The fundamental unit of the human brain is the neuron, which underlies the ability to memorize reason based on past experiences. Neuron networks are formed of these cells. These cells, in general, take input from various sources, execute non-linear neural activity, and provide completed output. ANNs are used in engineering applications in the same manner in other disciplines [28]. In ANN (Figure 3), the neuron is the unit where information is processed, which consists of:

(a) A signal *x_m_* in the input *m* of a synapse linked to neuron k is multiplied by synaptic weights *w_km_*;
(b) A linear combiner with an adder to total the inputs, which are weighted by the synapses of each neuron;
(c) The activation function *f*(.) is used to limit the amplitude of a neuron’s output. The normalized output amplitude range can be either [0,1] or [1,1];
(d) Bias, indicated by *b_k_*, is externally introduced; the purpose is to raise or reduce the overall impact of the activation function.

ANNs offer an interesting role in modelling soil behavior, especially the CBR value, which is affected mainly by the soil type and its associated properties [29,30]. ANN is equipped with the sophistication that allows the complex relationships between the various influencing soil factors (inputs) and the target soil properties (soaked CBR, unsoaked CBR, and UCS) to be revealed. It has the capabilities for these processes due to its ability for pattern recognition, data categorization, and regression, which allows the input variables to be analyzed, relationships to be found, and outputs to be determined. The processes go through iteration until there is a minimal difference between what was predicted by the ANN model and what was provided to the model for training. Structurally, the ANN algorithm was trained to perform an activation function through weights adjustment (connections values) between the neurons. The activation function generates the outputs computed from the sum of all the weights of each of the neurons ($W_{k}$) and the biases ($b_{k})$, with a constant value in place of the generalization errors of the neuron weights. The weight of the neuron, as shown in Equation (1), is calculated by summing the outcomes from the multiplication of each neuron connection’s weight and each input data approaching the neuron.

(1) $$W_{k\;}=\sum_{m=1}^{n}w_{km}x_{m}$$

where $x_{m}$ is the input *m* from the input layer and $w_{km}$ is the corresponding weight value of connections between neurons (*m*) at the input layer, and neurons (*k*) at the hidden layer, and those between the hidden and output layers. The activation function is used as part of the function variables to compute the sum of the neurons’ weights in the network to predict the output ($y_{k})$ as shown in Equation (2).

(2) $$y_{k\;}=f\;(W_{k\;}+b_{k})$$

For modelling neural network model, the Levenberg Marquadt backpropagation (LMBP) algorithm was employed. Three neurons in the input layer and one hidden layer comprising 10 neurons were used in the development of models. One neuron was used in the output layer for predicting the target variable.

#### 2.2.2. Random Forest Tree Model

The random forest tree (RFT) algorithm is an ensemble technique in which numerous decision trees are trained simultaneously using bootstrapping and aggregation; these operations are known as bagging. Bootstrapping is simultaneous training of multiple subsets of the training dataset using separate individual decision trees with different subsets of presented attributes. Bootstrapping is essential for ensuring that each decision tree in the RF is distinct, lowering the RF classifier’s overall variance. Because the RF classifier aggregates individual tree decisions for the final decision, it has a high level of generalization. With the danger of overfitting, the RF algorithm outperforms most other regression algorithms in terms of accuracy. In comparison with decision trees, RF does not require feature scaling, is more resistant to training sample selection and training dataset noise, and while its interpretation is difficult, hyperparameter adjustment is straightforward. For RF algorithm, each base learner (otherwise known as decision tree) are exposed to a variety of random feature vector subsets, which can be defined as $x=\left(x_{1},x_{2},\cdots ,x_{n}\right)$, where *n* represents the dimensional property of the vector for the decision tree. The goal at the end is to find the estimation function *h*(*x*) capable of estimating the *Y* parameter. Defining the estimation function, we have:

(3) L(Y,h(x))

where *L* is the loss function, whose purpose is to minimize the expected loss value. In regression and classification issues, the two functions defined in Equation (4) are the two most prevalent options.

(4) $$\begin{array}{c} L\left(Y,h\left(x\right)\right)=\left(Y-h{\left(x\right)}^{2}\right)\\ L\left(Y,h\left(x\right)\right)=I\left(Y\neq h\left(x\right)\right)=\{\begin{array}{c} 0,\;\mathrm{if}\;Y=h\left(x\right)\\ 1,\;\mathrm{otherwise}\; \end{array} \end{array}$$

With the basis learners coming together, the model’s ensemble is produced. Averaging is performed using Equation (5) for regression issues and Equation (6) for classification problems, with the base learners specified as $g_{1}\left(x\right),g_{2}\left(x\right),\cdots ,g_{j}\left(x\right)$


(5) $$h(x)=\frac{1}{J}\sum_{j=1}^{J}g_{j}(x)$$

(6) $$h(x)=arg\;max\sum_{j=1}^{J}I(y=g_{j}(x))$$

Rapidminer Studio (Version 9.8) was used for modelling the RF regression model. An operator “Select Attribute” and “Set Role” were used to distinguish input variables and the targets. The data were partitioned into the training and validation data in a ratio of (70/30) using “Split data”. “Optimal grid” was used to select the optimum hyperparameters, which comprises maximal depth of tree, number of trees, confidence, applies pre-pruning, random splits, and enables parallel execution and guess subset ratio. The optimization criteria such as minimal gain with linear grid scale ranging 0 to infinity was used. K-fold cross-validation was 10 number of folds, and the automatic sampling type optimized the performance. A total of 200 trees with a maximal depth of 20 yielded optimum results.

### 2.3. Evaluation Criteria

The statistical evaluation of the models was conducted using the slope of regression line obtained from experimental versus predicted plots, and the correlation coefficient (R) exists between the attributes and the target variable. Moreover, error indices, namely mean absolute error (MAE), root mean squared error (RMSE), and root squared error (RSE), were also used to assess the performance of the developed ANN and RF models [31,32,33,34]. The mathematical equations of the aforementioned performance indices are as below (Equation (7)–(9)):

(7) $$MAE=\frac{\sum_{i=1}^{n}\left|h_{i}-t_{i}\right|}{n}$$

(8) $$RMSE=\sqrt{\frac{\sum_{i=1}^{n}{\left(h_{i}-t_{i}\right)}^{2}}{n}}$$

(9) $$RSE=\frac{\sum_{i=1}^{n}{\left(t_{i}-h_{i}\right)}^{2}}{\sum_{i=1}^{n}{\left(\bar{h}-h_{i}\right)}^{2}}$$

where *h_i_* and *t_i_* are the *i*th actual and predicted output values, respectively; ${\bar{h}}_{i}$ and ${\bar{t}}_{i}$ are the average values of the actual and predicted output values, respectively; and *n* is the number of samples.

## 3. Results and Discussions

### 3.1. Unconfined Compressive Strength of CG

#### 3.1.1. Role of Lime

**Figure 4:**
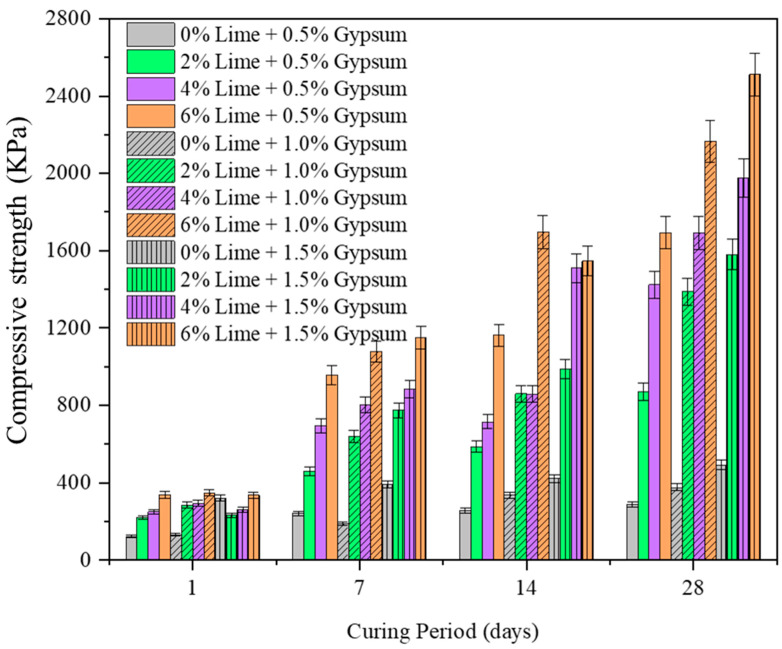
The variation in unconfined compression strength of coal gangue with curing period (1, 7, 14, and 28 days), lime (0 to 6%) and gypsum (0 to 1.5%) addition.

A thorough understanding of variation in UCS of CG with lime dosage takes paramount importance for its potential application as subbase material in pavements. The variation in UCS of CG with lime and gypsum is presented in Figure 4, and the influence of both the additives is evident with a considerable rise in UCS. With the increase in lime dosage, UCS linearly increased from 116 kPa (controlled case) to 320 kPa (6% lime addition), with an increment of 275% observed at a relatively lower curing period of 1 day. However, the greatest spike in UCS was noted for lime dosage of 2% and subsequent lime addition exhibited a relatively lower increments of 31% and 11% for 4% and 6% lime dosage. The initial spike in UCS, even at lower curing periods, may be attributed to the breaking of abundant silicon-oxygen bonds in CG followed by the formation of hydration compounds with the lime interaction [15]. Further, the pronounced effect of curing on UCS of CG is evident, with the highest jump of 211% observed for the rise in the curing period of 1 to 7 days. However, the increase in UCS at higher curing periods (14 and 28 days) is observed to be marginal, which may be attributed to the dissolution of alumino-silicates under (under lime induced alkaline environment) and subsequent precipitation of hydration compounds [15,35].

#### 3.1.2. Effect of Gypsum

The addition of gypsum further increased the UCS of lime treated CG with a more than 10 times increase (from controlled case) noted for 4% lime and 1.5% gypsum combination. The sustained and rapid increase in UCS of CG with gypsum addition is attributed to the alteration in the hydration reaction induced by the gypsum interaction with alumina of CG. The effect of curing is more pronounced with the gypsum addition, with more than 423% rise in UCS compared with 211% increment observed for the lime addition alone. The observed behavior is implicit with earlier observation of sulphate ions (in gypsum) reactions with aluminates (in CG) to form secondary hydrates of calcium alumina silicates (CASH). Similar observations were made by Ashfaque et al. [36] for the gypsum stabilization of lime-treated fly ashes [37]. Earlier researchers [15,37] have also noted that gypsum addition also eliminates the lime leachability phenomenon, which is generally observed in lime stabilization of soils [37,38].

### 3.2. CBR of CG

#### 3.2.1. Role of Lime

A comprehensive understanding of CBR is essential for the CG application as subbase material in pavements. The CBR of the lime stabilized CG is shown in Figure 4. From the results, it can be noted that there is a substantial increase in CBR of CG with the addition of lime. For zero curing period, with lime addition, the unsoaked CBR value increased linearly from 19 (untreated) to 85 (4% lime). The maximum increase in CBR was observed for 6% lime addition with an increment of 445%. However, the highest jump in CBR from the untreated case was observed for 2% lime addition, with 350% rise from the untreated case. The rise in CBR for higher lime addition (4%) was relatively lower, with 95% increment. The initial rise in CBR at lower curing periods can be attributed to the formation of hydration compounds which increase the resistance to load applied (penetration) [39]. It is evident from the results that curing has a pronounced effect on CBR values of CG. Further, the highest CBR values were reported for 4% lime addition which is identical to observations made for maximum dry density and hydraulic conductivity values. The initial increase in CBR with lime addition can be attributed to the flocculation of CG particles into granular fractions, thereby inducing greater resistance to loading [40]. The higher resistance manifests in the form of an increase in CBR value. The subsequent decrease in CBR values at higher lime addition (6%) may be attributed to the non-availability of reactive silica to form hydration compounds. Further, it is worth noticing that the loss in CBR value for the soaked conditions is relatively greater for a higher curing period.

#### 3.2.2. Effect of Lime and Gypsum

**Figure 5:**
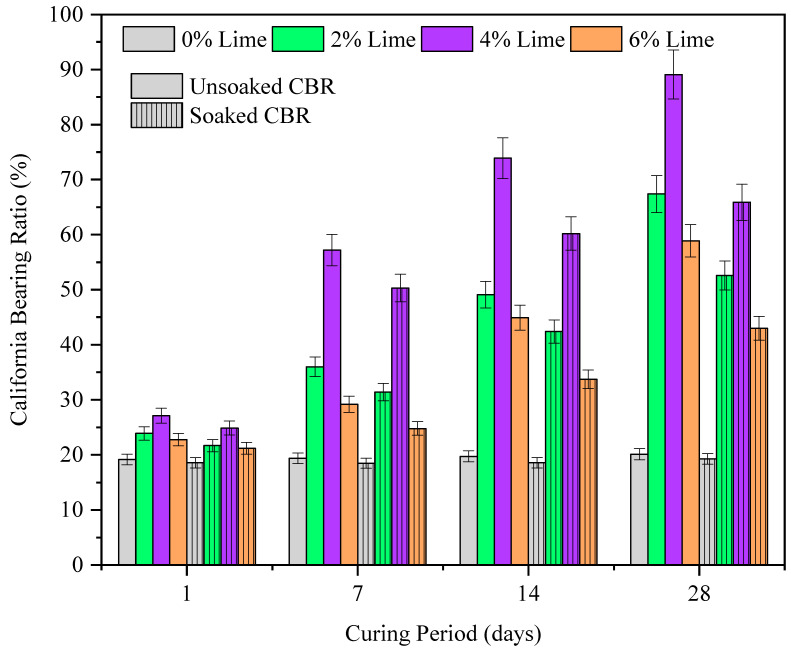
The variation in California bearing ratio of coal gangue with curing period (1, 7, 14, and 28 days) and lime dosage (0 to 6%).

**Figure 6:**
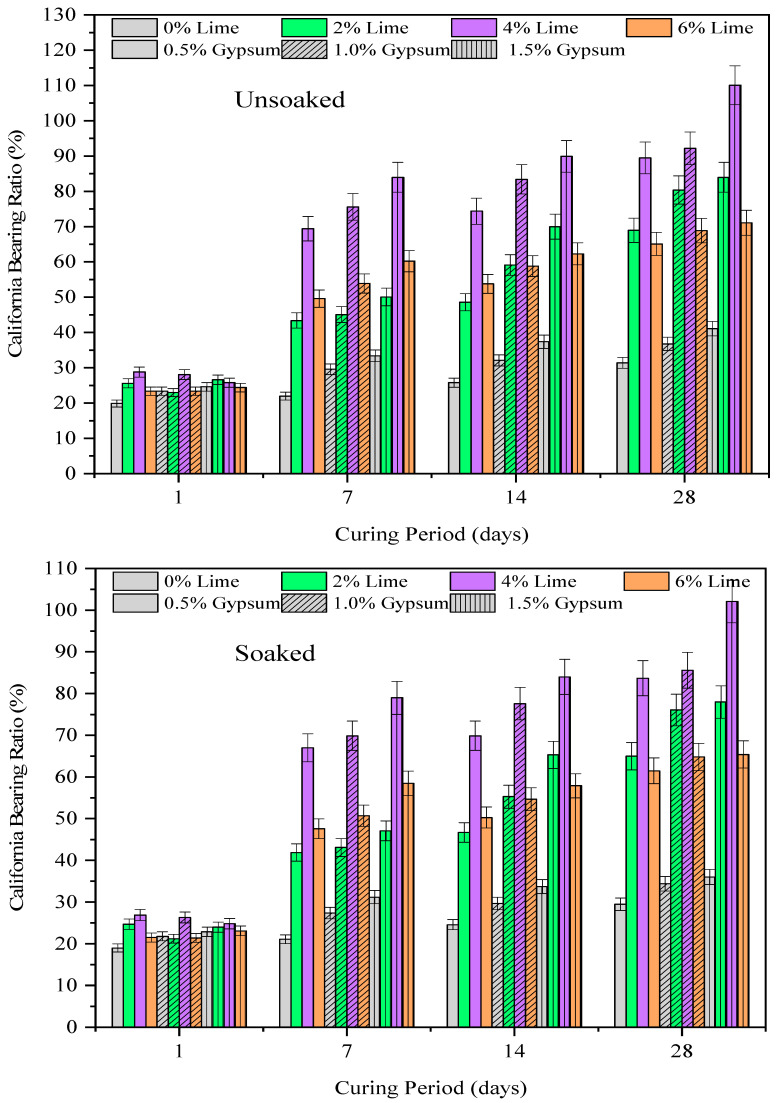
The variation in California bearing ratio (unsoaked and soaked) of lime treated coal gangue with gypsum and curing period up to 28 days.

The variation in the CBR value of lime treated CG with lime and gypsum is presented in Figure 5 and Figure 6, respectively. From the results, it can be observed that the CBR value of lime treated CG exhibited an increasing trend with the addition of gypsum, with the highest increment of 543% (compared with the controlled case) observed for 4% lime addition. Unlike UCS, the CBR value exhibited an optimum dosage of 4% lime which remained constant even with gypsum addition. Apart from the observations made for UCS, i.e., formation of hydration products, the sample prepared in CBR is substantially higher, which is primarily attributed to dual stage of CBR testing, i.e., (dynamic compaction with greater compact effort and loading stage). The amorphous silica subsequently reacts with available hydroxyl ions (with lime addition) to form hydration compounds in the form of calcium silicate hydrate (CSH). The hydration compounds reduce the pore spaces resulting in enhanced resistance to the applied load. The increased pattern observed with the lime addition remained consistent for varying curing periods, and understandably, with the rise in curing period, the hydration reactions proceed more rigorously, resulting in a further increase in CBR. The initial increase in CBR at a lower curing period (7 days) can be attributed to the onset of pozzolanic reaction [15,41]. The hike in CBR even at lower lime content (2%) is due to the increase in the solubility of silica caused by the breaking of Si-O and Al-O bonds by hydrated lime (Ca(OH)_2_), which subsequently reacts with hydroxyl ion (with lime addition) and reactive sulphate ions to form secondary hydration compounds [11,41]. The hydration compounds reduce the pore spaces resulting in enhanced resistance to the applied load. The optimum increase in CBR was noted at 4% lime addition and lime dosage beyond optimum exhibited a gradual rise in CBR, which is either due to the non-availability of reactive silica in CG to react with excessive free lime or completion of interaction between reactive silica and lime [41]. The increased pattern observed with lime addition remained consistent for varying curing periods, and understandably with the rise in curing period the hydration reactions proceeded more rigorously resulting in a further increase in CBR. The initial increase in CBR at lower curing period (7 days) can be attributed to the onset of pozzolanic reaction.

### 3.3. Prediction Performance of the Proposed Models

**Figure 7:**
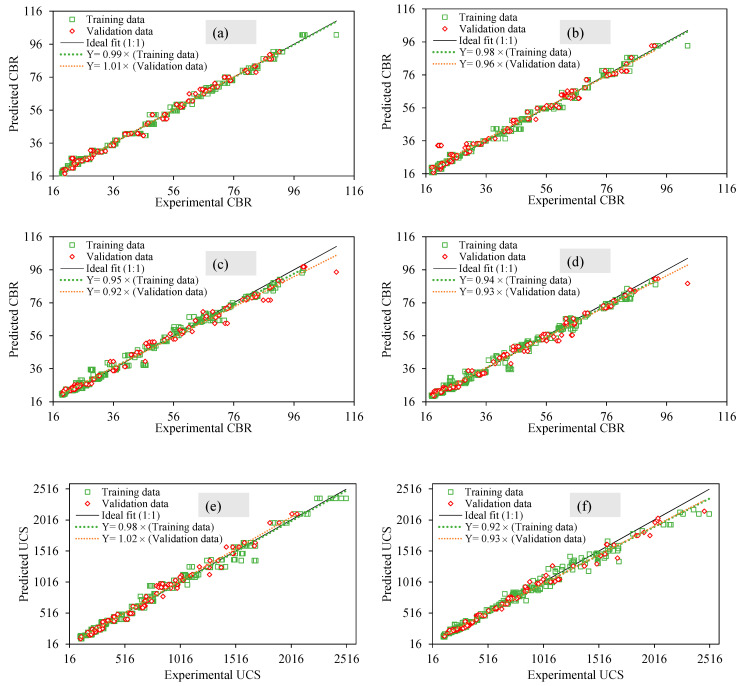
Statistical evaluation of: (**a**,**c**,**e**) ANN unsoaked CBR, soaked CBR and UCS, (**b**,**d**,**f**) RF Unsoaked CBR, soaked CBR and UCS, respectively.

Figure 7 depicts the experimental versus predicted results of the unsoaked CBR, soaked CBR, and UCS of the chemically stabilized CG by deploying ANN and RFT approaches, respectively. The regression slopes of training and validation datasets are compared for all the three characteristics using the two AI methods. All the regression slopes exceed 0.92 which depicts the accuracy of the formulated models using ANN and RF techniques. While determining the unsoaked CBR, soaked CBR, and the UCS values, the performance of the ANN models is recorded to be superior for both the training and validation datasets in contrast to the RF regression approach. This is because the regression slopes decrease by 1.01% (unsoaked CBR), 1.05% (soaked CBR), and 6.12% (UCS) in the case of training data modelled using ANN and RF approaches (Figure 7a–f). On the contrary, a decrease of 4.95% (unsoaked CBR), increase of 1.09% (soaked CBR), and significant decrease of 8.82% (UCS) can be observed in the case of validation data, modelled by the ANN and RF methods (Figure 7a–f).

Table 4 shows different performance measure indices (i.e., R, RMSE, MAE, and RSE) values to evaluate the unsoaked CBR, soaked CBR, and UCS using the ANN and RF approaches. It can be observed that all the R values exceed 0.990 which suggests the better performance of the two AI models. In the case of training and validation data, the R values in both the models are almost equal, with the exception of the soaked CBR model using the ANN method (R_training_ = 0.995 and R_validation_ = 0.990). Similar trend of RMSE values can be observed with exception of UCS modelled using the ANN and RF method (RMSE_training_ = 65.96 and 60.54, RMSE_validation_ = 71.67 and 68.659), respectively. The MAE and RSE values further validate the prediction performance of both the models with ANN approach outperforming the RF generated models to evaluate the unsoaked CBR, soaked CBR, and UCS. Note that the prediction performance follows the order: Unsoaked CBR > soaked CBR > UCS.

**Figure 8:**
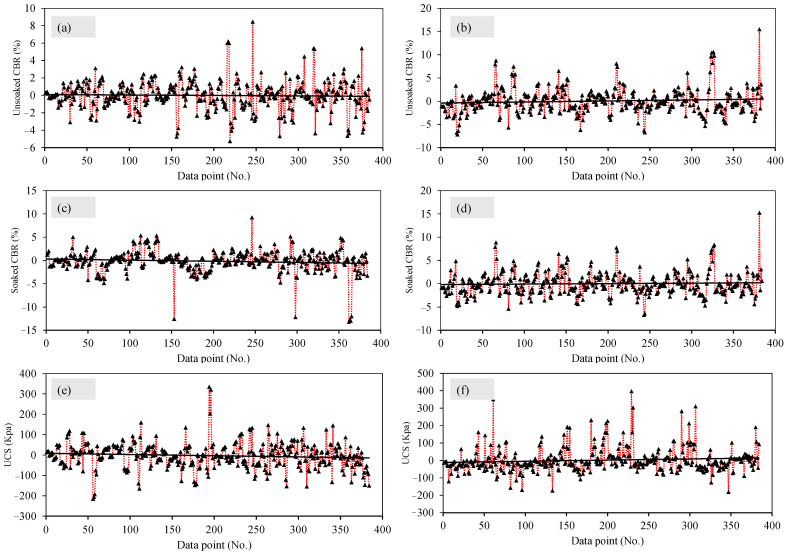
Residual errors plotted between experimental and predicted results in the case of: (**a**,**c**,**e**) ANN model, (**b**,**d**,**f**) and RF Model.

Figure 8 depicts the plots of residual errors for the entire dataset to evaluate unsoaked CBR, soaked CBR, and UCS, using the ANN and RF methods. It is already observed from the Figure 7 and Table 4 that the ANN approach is superior. Firstly, Figure 8a reveals that, up to 200 datapoints the (residual error values of) unsoaked CBR ranges between ±3.5 except a datapoint (at 150) where the output value approaches −4.2, in the case of ANN modelling. The datapoints beyond 200 are observed to be located between +8.2 and −5.5. On the contrary, Figure 8b depicts that the unsoaked CBR ranges between ±7 up to 300 points, whereas for the datapoints beyond it are between +16 (more conservative) to −6, in the case of the RF modelling. Secondly, Figure 8c exhibits a similar trend as that of Figure 8a such that the (residual error values of) soaked CBR ranges between ±5 except a datapoint (at 150) where the output value approaches −12, in the case of ANN modelling. The datapoints beyond 200 are observed to be located between +9 and −12. In comparison, the Figure 8d, i.e., RF modelled soaked CBR, all the output values range between ±7.5 except for one last datapoint which is yielded as +15. Thirdly, Figure 8e reveals that, up to 200 datapoints (residual error values of), the UCS ranges between +180 and −210, in the case of ANN modelling. The datapoints beyond 200 are observed to be located between ±120. In contrast to the ANN modelled UCS values, the RF modelled UCS (Figure 8f) depicts that all the datapoints have a residual error ranging from +400 to −190 such that most of the points are positive thus showing that RF predictions are relatively more conservative. Hence it can be concluded that, the ANN models exhibit better performance than the RF approach in terms of regression slopes, error indices, and residual error plots. However, both the AI models possessed higher a degree of accuracy and can be applied where necessary.

While comparing the performance of other AI models for stabilized soil mixes, it was revealed that the support vector machine (SVM) model prediction performance is significantly better than the traditional fitting function [42]. In another study a comparison was made between the results obtained by the prediction techniques. To evaluate the robustness of the applied methods, the 5-fold cross-validation (CV) was deployed. It was found that the AI methods are capable of predicting the UCS. Overall, Gaussian process regression (GPR) exhibiting R^2^ of 0.9955 and an RMSE of 0.52169, showed excellent performance. Lastly, the UCS prediction intelligence methods were ordered as GPR, decision tree (DT), support vector regression (SVR), long short term memory (LSTM), deep neural networks (DNN), and K-nearest neighbor (KNN) [43].

### 3.4. Model Validity

#### 3.4.1. First Level Validation

The efficacy, robustness, and comparative analysis of the proposed ANN and RF models in predicting CBR (soaked and unsoaked) and UCS of the chemically stabilized CG is assessed with the aid of the regression line slope of the statistical metrics (R, RMSE, MAE, and RSE). The ratio of experimental records (384 datapoints) to explanatory input variable (three input variables are considered in this study) must not be less than three, and should ideally be greater than five in order to create an efficient machine learning (ML) model [44,45]. For this study, it becomes 128, which satisfies that ratio requirement beyond the acceptable limit for an effective and efficient ML model for the chemically stabilized CG, signaling a reliable ML model. In validation stages, the actual (experimental) and estimated UCS results for stabilized CG, as well as their performance indices, are displayed (i.e., slope, R, MAE, RSE, and RMSE). The ideal fitted line with a slope of 1 is depicted by the 45° standard regression line. The displayed points’ distribution must be closer to the standard line, have a slope approaching 1, R > 0.8, and have small error metrics for great performance and strongly linked models (MAE, RSE, and RMSE). For the ANN model, the regression line slope of the unsoaked CBR and soaked CBR corresponds to 1.01 and 0.92, respectively, for the validation stages. For the RF model, slope of the unsoaked CBR and soaked CBR corresponds to 0.96 and 0.93, respectively, for validation data. Similarly, for the ANN and RF models, the slope of the UCS corresponds to 1.02 and 0.93, respectively, for validation data. The high values of the slopes reveal strong correlations between the experimental and model-predicted outputs validation stage. Similar to the slope values, R-values of the validation stages are almost equal to the R-values of the training stages for the soaked CBR, unsoaked CBR, and UCS, which ruled out the possibility of data overfitting of the finally proposed models.

Though the slope and R-value are good indicators of an efficient and effective predictive model, they are not exclusive indicators to assess the robustness of the trained ML models. Other statistical error indices such as MAE, RSE, and RMSE were also considered in this study, to confirm the veracity of the proposed ANN and RFT models. Generally, in both ANN and RF models, the error indices for the unsoaked CBR and soaked CBR are much lower than those of UCS. Judging by performance via the error indices, the proposed ANN model performed relatively better than the proposed RF model. For the training data, the ANN model was better with lower in two of the three error indices (MAE and RSE), and in the validation data the ANN model was relatively better with two of the three error indices (RMSE and RSE).

#### 3.4.2. Second Level Validation

Computational ML models are widely recognized for overfitting during the training phase [23,46], resulting in good performance for both training and test datasets. However, such models may produce unexpected findings for samples collected from various experimental settings. As a result, the developed model must be confirmed using previously unexplored datasets [47]. Parametric analysis was performed on a simulated dataset. Simulated data were created such that one of the three variables was changed in between its extremes in equal offsets whereas the other parameters were maintained at their average values. Subsequently, the 2nd variable changed whereas the 1st and last one were kept constant at their average magnitudes. The simulated dataset was tested using the trained model developed for unsoaked CBR, soaked CBR, and UCS values. As illustrated earlier, the ANN model performed better compared with the RF model, therefore test data were analyzed using the ANN model in the MATLAB 2021 environment.

**Figure 9:**
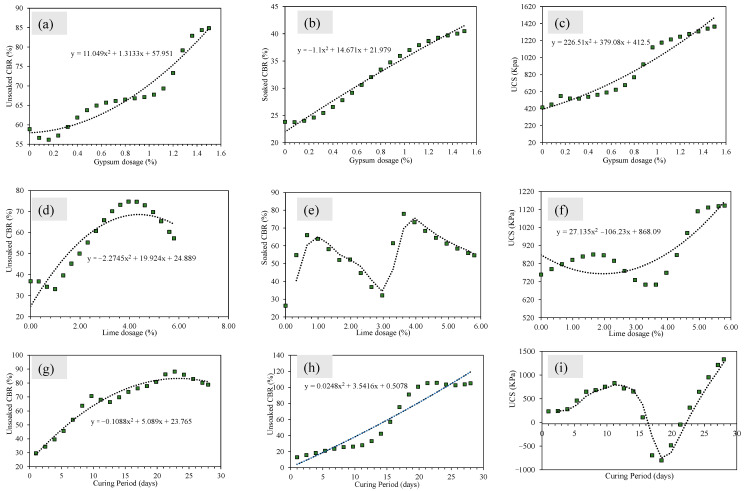
Parametric or monotonicity analysis of the developed ANN models for the simulated data of unsoaked CBR (**a**,**d**,**g**), soaked CBR (**b**,**e**,**h**), and UCS data (**c**,**f**,**i**).

Figure 9 displays the results of the parametric analysis. Figure 9a,d,g interprets that unsoaked CBR increased in the form of a 2° polynomial with the change in gypsum and lime dosage. The curing period also increased the magnitude of unsoaked CBR. Lime dosage up to 1.5% yielded the maximum CBR, whereas gypsum content beyond 4% reduced the CBR value. For soaked CBR, the magnitude linearly increased with the rise in gypsum dosage, and 1% and 4% lime dosage yielded the maximum CBR value. The curing period also increased the magnitude of the CBR linearly. The magnitude of UCS is also interpreted an increasing trend with an increase in gypsum and lime dosages.

## 4. Conclusions

In the present study, artificial neural network (ANN) and random forest (RF) regression-based prediction models were developed to demonstrate the variation in the UCS and CBR values of coal gangue (CG) stabilized using calcium-rich lime and gypsum. The following conclusions were drawn from the study:

- The stabilization of CG resulted in a linear increase in the UCS of CG, with the highest increment (10×) noted for the lime and gypsum dosages of 6% and 1.5%, respectively. In contrast to the strength behavior, the CBR value of CG attained optimum value at 4% lime dosage, and the subsequent addition of gypsum resulted in a further increase in CBR values;
- Levenberg Marquadt backpropagation with 10 neurons in the hidden layer yielded the optimal performance of the formulated ANN model. On the contrary, among several iterations in the form of optimization grid for developing RF regression model, the minimal gain criteria with 200 trees and a maximum depth of 20 manifested the best performance of the RF model;
- The performance of the developed models were evaluated using the slope of regression lines compared with the ideal slope, correlation values (R), and error indices, namely MAE, RMSE, and RSE. For the ANN model, the training set yielded the minimum correlation value (R = 0.993) whereas for soaked CBR and unsoaked CBR, R = 0.995 and 0.997, respectively. Furthermore, the values of MAE for the training sets were recorded as 45.98 kPa, 1.180%, and 1.409% for the UCS, unsoaked, and soaked CBR of chemically stabilized CG, respectively;
- The RF regression model reflected slightly lower accuracy in terms of R-values and error indices for the case of the training sets. The lowest value of R was observed comparable to the ANN model whereas the highest R was noted as 0.995. Similarly, the MAE values for the UCS, unsoaked, and soaked CBR equaled 46.95 kPa, 1.81%, and 1.77%, respectively;
- The developed ANN and RF models were tested using two-stage validation. In the first stage, an unused 30% of the dataset was utilized. The values of R and error indices reflected comparable performance to that of the training data. It was also seen that all these values were close to the training data, suggesting no overfitting issues in the developed models. In the 2nd stage of validation, the ANN model was employed to assess the impact of contributing parameters on the target variables (UCS, unsoaked CBR, and soaked CBR). In addition, a parametric analysis was conducted which showed 1.5% gypsum and 4% lime dosage levels as optimum for yielding maximum unsoaked and soaked CBR values; whereas the values of the optimum UCS were achieved at 1.5% gypsum and 6% lime. These results are in accordance with the experimental study conducted here and are also corroborated in past literature.

## Figures and Tables

**Table 1:** Properties of coal gangue acquired from Bhupalpally, Telangana, India (controlled case).

Property	Value	ASTM Standard
Specific Gravity	2.57	ASTM D854
Ph	7.2	ASTM D4972
UCS Classification	SP	ASTM D2487
Liquid Limit (%)	28	ASTM D4318
Plasticity Index (%)	NP	ASTM D4318
Optimum Moisture Content (%)	17	ASTM D698
Maximum Dry Density (g/cc)	1.90	ASTM D698
Unconfined Compressive Strength (kPa)	116	ASTM D2166
California Bearing Ratio (soaked, %)	18	ASTM D1883

**Table 2:** Chemical composition of coal gangue by using X-ray Fluorescence (XRF) analysis.

Chemical Constituents	Value
Silica (SiO_2_)	52.70
Alumina (Al_2_O_3_)	22.60
Ferric oxide (Fe_2_O_3_)	6.37
Calcium oxide (CaO)	3.45
Magnesia (MgO)	1.72
Titanium (TiO_2_)	0.98
Potash (K_2_O)	2.68
Sodium oxide (Na_2_O)	0.75
Sulphur (SO_3_)	0.53
Loss on ignition	8.22

**Table 3:** Details of the input and target variables as well as the database used in ANN and RF modelling.

S. No	Input Variables Used in This Study			No. of Samples Tested for UCS and CBR (Target Variables)
	Gypsum Dosage (%)	Lime Dosage (%)	Curing Period (Days)	
1	0	0	1	6
2	0	0	7	6
3	0	0	14	6
4	0	0	28	6
5	0	2	1	6
6	0	2	7	6
7	0	2	14	6
8	0	2	28	6
9	0	4	1	6
10	0	4	7	6
11	0	4	14	6
12	0	4	28	6
13	0	6	1	6
14	0	6	7	6
15	0	6	14	6
16	0	6	28	6
17	0.5	0	1	6
18	0.5	0	7	6
19	0.5	0	14	6
20	0.5	0	28	6
21	0.5	2	1	6
22	0.5	2	7	6
23	0.5	2	14	6
24	0.5	2	28	6
25	0.5	4	1	6
26	0.5	4	7	6
27	0.5	4	14	6
28	0.5	4	28	6
29	0.5	6	1	6
30	0.5	6	7	6
31	0.5	6	14	6
32	0.5	6	28	6
33	1	0	1	6
34	1	0	7	6
35	1	0	14	6
36	1	0	28	6
37	1	2	1	6
38	1	2	7	6
39	1	2	14	6
40	1	2	28	6
41	1	4	1	6
42	1	4	7	6
43	1	4	14	6
44	1	4	28	6
45	1	6	1	6
46	1	6	7	6
47	1	6	14	6
48	1	6	28	6
49	1.5	0	1	6
50	1.5	0	7	6
51	1.5	0	14	6
52	1.5	0	28	6
53	1.5	2	1	6
54	1.5	2	7	6
55	1.5	2	14	6
56	1.5	2	28	6
57	1.5	4	1	6
58	1.5	4	7	6
59	1.5	4	14	6
60	1.5	4	28	6
61	1.5	6	1	6
62	1.5	6	7	6
63	1.5	6	14	6
64	1.5	6	28	6
	Total number of samples			**384**

**Table 4:** Statistical evaluation of the developed ANN and RF models.

Model	Parameter	Training Data				Validation			
		R	RMSE	MAE	RSE	R	RMSE	MAE	RSE
ANN Model	Unsoaked CBR	0.997	6.940	1.180	0.005	0.996	6.866	1.322	0.001
	Soaked CBR	0.995	6.570	1.409	0.010	0.990	6.740	2.039	0.004
	UCS	0.993	65.96	45.980	0.014	0.993	60.54	46.792	0.005
RFT Model	Unsoaked CBR	0.995	6.874	1.806	0.012	0.993	7.009	2.267	0.004
	Soaked CBR	0.994	6.563	1.770	0.013	0.993	6.715	1.976	0.003
	UCS	0.993	71.67	46.955	0.018	0.994	68.659	45.522	0.006

## Data Availability

The data used in the development of AI models can be furnished upon suitable request from the corresponding author.
